# Study protocol: exploring water intake and hydration status changes in young children in a day care center in different climate scenarios

**DOI:** 10.1186/s12887-025-06271-7

**Published:** 2025-11-28

**Authors:** Aziza J. Belgardt, Hermann Kalhoff, Mathilde Kersting, Kathrin Sinningen, Benjamin Bechtel, Thomas Lücke

**Affiliations:** 1 Research Department of Child Nutrition, University Hospital of Pediatrics and Adolescent Medicine, St. Josef-Hospital, Ruhr-University Bochum, Bochum, Germany; 2 Pediatric Clinic Dortmund, Dortmund, Germany; 3 Bochum Urban Climate Lab, Ruhr-University Bochum, Bochum, Germany; 4 University Hospital of Pediatrics and Adolescent Medicine, St. Josef-Hospital, Ruhr- University Bochum, Alexandrinenstrasse 5, Bochum, 44791 Germany

**Keywords:** Water intake, Dietary guidelines, Hydration status, Heat stress, Climate change, Young children

## Abstract

**Background:**

Global climate change causes rising of the average temperature as well as an increased number of days with extreme heat, even in the temperate climate of Germany. Children are a particularly vulnerable group according to heat stress and consequentially water loss. Even small changes in the water balance can lead to functional impairments. Therefore, quantification of exact water loss as well as the knowledge of hydration status changes in children under heat stress are important and can contribute to renewing water intake recommendations in accordance with climate change. This may promote approaching public health issues.

**Methods:**

In this study water loss, hydration status data and voluntary water intake of children are collected. Therefore, 3–6 years old children from a German kindergarten are recruited. Weight, urine osmolality and saliva osmolarity are measured twice a day on two different testing days with different temperatures (day 1: hot summer day with heat peak; day 2: fall-like day with neutral temperatures). In-between the tests on each day, the children are exposed to these climate scenarios by playing outside for 2 h just like the usual daily routine. The drinking amount as well as the activity during the observational time is recorded. Additionally, the collected data is compared to previously published data on computed estimation of body core temperature changes and water loss in heat on a theoretically modelled 3-year-old child.

**Discussion:**

The weight difference before and after exposure (in dependence of the water intake), provides information about the water loss (thermoregulation) in the observation period. The comparison of newly collected data with modelled water loss can elaborate variations in climate and physiological responses and provide information about, whether the model calculation can be transferred to German conditions. Furthermore, hydration status changes, as reflected by the urine osmolality, are expected to be different between the two test days depending on the extent to which the higher water loss through sweating is compensated by different water intake through voluntary drinking. By means of the collected data, drinking recommendations for children may be aligned and specified in accordance to different climate scenarios.

**Trial registration number:**

German Clinical Trials Register (Deutsches Register Klinischer Studien), DRKS00033942,19th August 2024.

## Background

Global climate change is a multidimensional public health issue and a threat to the environment [1]. The rate of temperature rising has lately increased immensely [1]. This global warming causes reduced food and water security [1]. Presently, approximately half of the world’s population suffers from water scarcity for at least one season throughout the year [1].

Climate change impacts the human well-being directly [2, 3]. Especially in urban areas local heat extremes and air pollution are intensified [1, 2]. In metropolitan areas the temperature tends to be higher in comparison to rural surroundings, due to locally human-caused heat-production/accumulation [1, 2]. This effect is referred to as urban heat islands [2].

Urban heat in general can have various negative effects on the population’s health outcomes, such as increased mortality and morbidity, mental health issues as well as generally increased emergency room and hospital visits due to heat-related illnesses [3, 4]. This includes heat exhaustion, heat injury and heat strokes [5]. The risk might be reduced by staying hydrated.

### Thermoregulation

The human body is capable of maintaining a nearly constant body temperature even at different ambient temperatures. Facing heat, the main mechanisms to prevent overheating of the body are: increase in vasodilation (heat is transported away from the body center towards the skin and consequently to the environment) and sweat production [3, 5]. These primary ways are also defined by the term active thermoregulation. The passive thermoregulatory system compromises body constitutions like body height, which affects heat exchange [5]. Exposure to high ambient temperatures leads to an increase in sweat production and therefore water loss.

Heat stress refers to the physical status of the human body when the natural thermoregulation cannot obtain heat balance; heat accumulates inside the body and causes a rise in body temperature [2, 5]. Heat stress occurs due to environmental conditions, metabolic heat production and clothing [2, 5]. Under heat stress the sweat production reaches a maximum. Water loss may be immense. Sufficient fluid intake is therefore particularly important in hot temperatures to compensate for water loss through sweating [6].

Thus, with increasing ambient temperature, the already prominent importance of water as the most important nutrient becomes even more pronounced [6, 7].

### Water: the most important nutrient

The human body consist of a high percentage of water. Depending on age, it ranges from 80% in newborns to 35% in the elderly. The daily turnover rate of water is high [8, 9]. The hydration status describes the dynamic balance between fluid intake and water loss. Even small deficits of water are associated with headache, poorer physical and cognitive performance as well as other impairments [10, 11].

Several intrinsic factors can influence the temperature regulation during heat stress [5]. Due to their higher body water percentage and other body constitutions included in the passive thermoregulatory system, children are a particularly vulnerable group to dehydration [6, 8, 12, 13]. Previous findings suggests, that children’s higher body surface-to-mass-ratio causes faster skin and core temperature rise and consequently a higher water loss-to-weight-ratio [12–14]. Furthermore, children generally require a higher water intake in relation to their body mass in comparison to adults [6, 15, 16]. Unfortunately, cross-sectional nutrition surveys suggest that worldwide children’s fluid intake is below current recommendations [17].

### Call to action

As global warming increases, adapting drinking habits are becoming more important [6]. About 559 Million children worldwide currently face a high frequency of occurring heat waves, with an expected rising trend in the upcoming years [18]. Even people living in the temperate climate zones (e.g., Germany) are at great risks of voluntary dehydration [19].

Drinking recommendations therefore need to be updated for different weather scenarios, especially for high temperatures [6]. Since children are a particularly vulnerable group, the planned study described with this study protocol, will provide new knowledge about water loss under heat exposure in young children and therefore contribute to developing weather-related drinking recommendations.

The planned observational study will take place in a kindergarten in Bochum, Germany. Bochum, being a part of a big conurbation named “*Ruhrgebiet*”, meets the conditions for an urban heat island, as well. Young children from this kindergarten are recruited to collect new data on hydration status changes and quantification of water loss in different climate scenarios in urban heat islands.

The newly obtained data on the water balance of young children in the heat can be compared with the published results from model calculations on water loss through sweating in children of the same age group. These new perceptions might allow modifying recommendations for fluid intake in young children during heat waves. On the long term, these findings may also help to improve the increasing occurrence of heat-related illnesses and general wellbeing and therefore addresses a highly relevant public health issue.

## Methods

### Aim of the paper

Using a small-scale observational field-study under everyday conditions, the main aim is to investigate the impact of heat stress and activity on the hydration status of young children aged 3–6 years and quantify the amount of water loss through sweat for the first time. The collected data can provide knowledge for the need to adjust water intake under heat stress and might contribute to adapting drinking recommendations for children depending on different weather scenarios.

Furthermore, the voluntary water intake in children in hot and neutral temperatures is quantified and related to the hydration status. These findings might provide information for developing adequate drinking promotion actions for children and contribute to public health.

In addition, findings are compared to previously published data on computed estimation of water loss in heat on a theoretically modelled 3-year-old child. This comparison can elaborate variations in climate scenarios in Germany and Japan and their impact on children’s thermoregulation. The results of the model might add to the knowledge of physiological responses under heat stress in another climate scenario.

Furthermore, the salvia osmolarity is taken from the young subjects. This no-invasive technique is a new measurement approach for determining hydration status. So far, this method hasn’t been validated adequately for the use on children. In addition to the above-described study this nested feasibility study shall investigate the use of salvia osmolarity in pediatrics.

### Objectives

This study examines three hypotheses in the primary (confirmatory) hypothesis of this paper it is expected, that measured water loss (body weight difference, corrected for fluid-intake) is higher in 3–6-year-old subjects when exposed to heat for 2 h intraindividual compared to exposure to neutral temperatures at the same duration.

Secondly, it is expected, that the water loss calculations of the computed model of a 3-year-old child in Japan is suitable for comparison to German conditions. Last, it is expected that children (aged 3–6 years) show a greater hydration status change under heat exposure compared to a neutral tempered environment. This shall be tested by commonly used, non-invasive clinical parameters (weight change in kg, urine osmolality in mosmol/kg) (Table 1).

**Table 1:** Overview of the study characteristics

Category	Information
Title	Water intake and hydration status changes in young children in a day care center with locally collected microclimate data in different climate scenarios: An observational study
Registration number	DRKS00033942
Ethic committee	Ethic-Committee Westfalen-Lippe Münster, Germany
Identification code	2024-304-f-S
Date of approval	16th August 2024
Study type	Small scale observational field-study - Monocentric - Prospective, experimental - Uncontrolled, open-label - One study arm
Location	Kindergarten in Bochum, Germany
Duration	Approx. 1 Month - 2 test days each with 2 h observation
Subjects/participants	Children, aged 3–6 years
Sample size	Approx. 30 participants
Inclusion criteria	-Registered child of the recruited kindergarten -Generally healthy -Basic skills in German language -Completed declaration of consent of the caregivers
Exclusion criteria	-Acute or chronic disease, particularly those with specific need for a good hydration: diabetes, cystic fibrosis, diseases of kidney, liver, cardiac diseases, acute/chronic diarrhea, vomiting, acute/chronic respiratory disease, fever -Generally sensible against heat
Study hypothesis	*Primary hypothesis*: Measured water loss (weight difference, corrected for fluid-intake) in 3–6-year-old subjects is higher at 2 h exposure to heat compared to an exposure to neutral temperatures at the same duration. *Secondary*: The computed model of a 3-year-old child is suitable for comparison to the measured water loss (body weight change) in 3–6-year-old participants exposed hot weather at the same duration. *Secondary*: Children (aged 3–6 years) show a greater hydration status change, measured with common parameters (weight, urine osmolality) under heat stress compared to a neutral tempered environment.
Endpoints	*Primary*: body weight (kg) *Secondary*: Urine Osmolality (mmol/kg), salvia Osmolarity (mosmol/liter), Amount of water intake (ml), physical movement, age
Study design	Two test days with different ambient temperatures; measurement of hydration status change and water loss of children playing outside

### Study design and setting

This small-scale observational field-study is planned as a two-test day survey to provide data of the impact of different temperatures on hydration status change and water loss in young children. This monocentric study is underway by recruiting a kindergarten with an outdoor area in Bochum, Germany. For the data to be valid, the climatic conditions during the measurement period must be the same for all participants. Different locations of kindergarten can already show huge climatic differences even within the same model city which justifies the monocentric design. Using one study arm, approx. 30 children are included to participate at both days. Therefore, this study is uncontrolled and open-labeled. Using a follow-up with the same subjects helps to minimize interindividual variability and provides meaningful data for the comparison of changes in weight, urine osmolality, saliva osmolarity and drinking behavior between two weather scenarios. The test days are chosen between June and August 2025 based on weather forecast data and scheduled at least two days prior with the educators/parents of the kindergarten.

The Japanese weather scenario of the model calculation published in 2019 [13] is used as a starting point for setting the first days weather condition. To provide preservation for possible health outcomes and due to potential climatic differences in the subtropical climate compared to the temperate climate of Germany the temperature-range is set lower (Table 2). The test day shall have daily T_max_ of approximately 30 °C with dry heat and at least nearly clear sky for potential, periodic sun exposure. On the second test day the weather shall provide neutral, pleasant climate with daily T_max_ approx. 22 °C and no rain (Table 2).

**Table 2:** Comparison of weather data of computed calculation and possible weather scenario in Germany

Weather data of the model calculation	Realistic expectation of weather data of warmer test day
35.9–36.5 °C Approx. 34–46% humidity Sunny side/shady side of a road Approx. 0.2–4.5 m/s wind	Approx. daily T_max_ 30 °C 30–50% humidity Sunny, few-no clouds Not windy

**Fig. 1:**
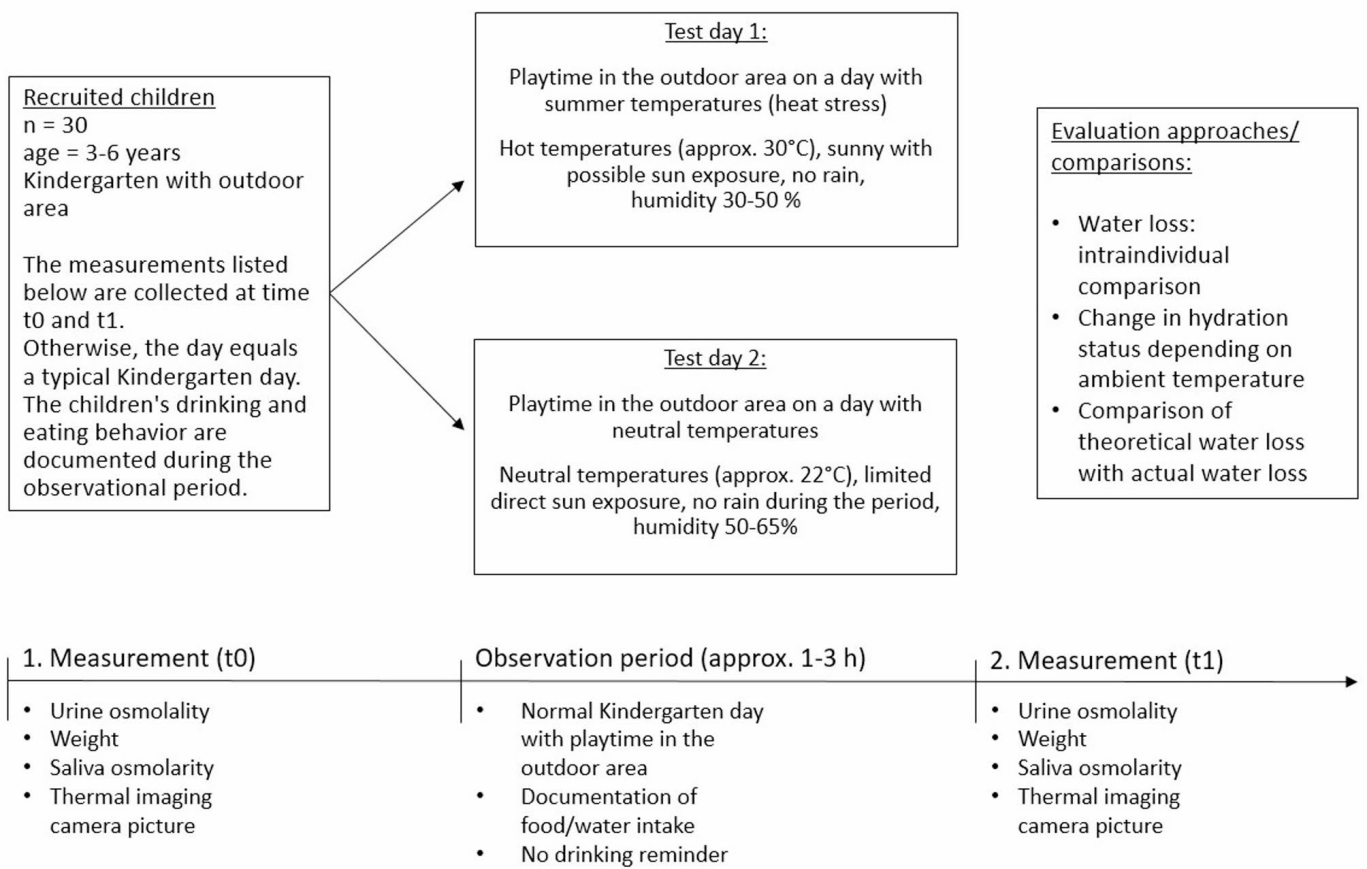
Flow chart of the observational study

The test day is comparable to a regular day at the kindergarten. The children are dropped off in the morning until approx. 9 o’clock. Children who haven’t eaten at home are offered breakfast at the kindergarten. They are free to eat and drink *ad libitum*. Especially in summertime most of the Kindergarten in Germany provide “play time outside” in the mid-morning before lunch. At least 15 min after breakfast the first set of data is collected (t0: urine osmolality, weight, saliva osmolarity). The movement measurement device is attached to the children’s clothes. Afterwards the children play outside and the observational period starts. The playtime lasts from approx. 10 o’clock until 12 o’clock. Water is provided in personalized cups. Children drink self-directed. In case a child needs to use the bathroom during the observational phase, the child is weighed right before and after the bathroom use to quantify the amount of urine excretion. After returning inside for lunch, the second data set is collected (t1: urine osmolality, weight) and the movement tracker is detached. After that the test day is over and the children are actively reminded to drink. Biometeorology conditions are monitored in shaded and sunny environment outside during the play time. The same procedure is repeated for the second test day with the same subjects in a different weather setting (Fig. 1). The two test days will take place within two weeks.

### Measurement methods

#### Weight

To provide the children’s privacy their weight is measured in a separate room. The weight is measured topless. The remaining clothes are noted to ensure that the children wear the same at the second measurement that day. The weight is measured by a calibrated personal scale. Scientists of the Research Department of Child Nutrition help the children and document the data.

#### Urine osmolality

Urine samples are collected twice at the test day. The children therefore pee in a potty or directly in a urine cup. Urine from the potty is decanted into a urine cup. The kindergarten teachers help the children by collecting the urine adequately.

Urine samples are cooled and transferred to a laboratory.

The alteration between t0 and t1 is calculated by the difference of the urine osmolality.

#### Drinking amount

Each child receives their own (sippy) cup filled with 250 ml water. In case they finish the cup, it is refilled with 250 ml. After the observation period, the amount of the water left in the cup is measured with a 1 ml-scaled measuring cylinder. The difference accounts for the drinking amount.

#### Thermal imaging camera

In addition, some participants have their picture taken with a thermal imaging camera before and after the observational period on both days. With the help of this imaging, the skin temperature change can be visualized. The Children therefore need to take off their shirt. The photography takes place in a separated room.

#### Saliva osmolarity

The salvia osmolarity is a new measurement approach for determining hydration status. The salvia osmolarity is measured by using the MX3 Hydration Testing System, Pro Version. The MX3 LAB is assembled with a test strip, which is held against the tongue of the child for approx. 3 s. The osmolarity can be read off the testing system right away.

#### Activity-tracker

The activity during the observation of the subjects is measured by the ActiGraph wGT3X-BT, which documents physiological total movements. This small device can be worn in several wear locations. It is fixed by belt in waist level of the participants, as recommended for young children.

#### Weather data

The weather data is collected by two ClimaVUE50 Compact Digital SDI-12 Weather Stations with Black Globe Temperature Sensors, which is installed in the open space of the kindergarten. The specific microclimatic weather data of both observational periods is collected and evaluated by the Bochum Urban Climate Lab of the Ruhr-University of Bochum, Germany.

All measurements are non-invasive and respect the privacy of the subjects. The children participate voluntarily and are allowed to refuse measurements. Furthermore, the measurements can be easily integrated in the everyday life in the kindergarten.

### Participants

The participants are aged 3–6 years of both sexes. To be eligible, children need to be a registered member of the kindergarten, own basic German language skills to communicate possible thirst and be in a generally good health. Furthermore, the child’s parents or caregivers must sign and return the written informed consent no later than the morning of the first test day.

Children, who have acute (e.g., cold) or chronic disease (e.g., cystic fibrosis), particularly those with specific need for a constant well hydration status, are excluded from participation (Table 1). Furthermore, caregivers are asked about general sensitivity against heat and participants might be excluded based on particular concerns about possible occurrence of heat-related illnesses. A clinical risk assessment was not performed since the study setting corresponds to a typical day in kindergarten with no further intervention.

### Recruitment

The participating kindergarten is picked based on specific interest in participation and motivation of the kindergarten teachers to help with collecting samples. The kindergarten is eligible, if an outdoor area is available. Furthermore, a kindergarten based inside the urban area of Bochum is preferred over those in the more rural peripheral area, to establish an approximate urban heat island scenario of the model calculation [13]. The recruiting takes place in cooperation with the responsible municipal department of the city of Bochum. On a parent-teacher conference the research-project firstly is introduced by the research team. Caregivers have the possibility to freely ask questions and receive informational material as well as the declaration of consent. These documents are put up on the notice board as well for parents who haven’t attended the parent-teacher conference. The documents include contact data of the research team for further possible question according to the study.

### Statistical analysis

Due to lacking preliminary data, a classical sample size calculation wasn’t possible. Sample size therefore was set based on the expected water loss amount from the modeled calculation for young children [13] and weight differences with standard deviation taken from older children [20]. The sample size estimation was supervised by a statistical expert team. The participant determination for this study was based on the primary hypothesis.

Even though water loss quantification due to sweating, especially in children, is basically important for determining adequate drinking recommendations, published data is rare so far. In Germany, current drinking recommendations are mainly based on mean 24-hour water intake in accordance with mean urine parameters in a cohort aged 4–7 years and 7–11 years [7]. To our knowledge the study planned here is the first attempt to quantify water loss based on weight change in young children between 3 and 6 years in the heat. Therefore, the calculation of an effect size based on former published data wasn’t possible.

The computer-based model calculation for the water loss through sweat of a 3-year old child under heat stress indicates a water loss of 142 g within 60 min [13]. Based on the calculated sweat rate of 3.85 g/min/m^2^ maximum and stated skin surface area, water loss of about 390 g within 3 h is expected in heat on the maximum sweat rate. As the sweat rate rises within approx. the first 20 min until it reaches a maximum, the sweat amount is expected to be slightly smaller in our kindergarten situation.

The paper Kojima et al. 2018 conducted a comparison in young adults, were the difference between computed water loss and measured water loss on humans equals 1,4 g (396.6 g and 398.0 g water loss in 90 min) but with no statement about the statistical significance of this comparison [21].

Since there are no experimental water loss data with standard derivation for children this age under heat stress available or for the modeled calculation in general, we included data for older children from the literature. Inbar et al. (2004) published data for boys aged 9.4 ± 0.6 years exercising on 50% of their max. oxygen uptake for 85 min (with rest in between sets) in a hotter environment than on our planned test day [20]. Whole-body sweating rate was calculated based on change in body mass. Total sweat production accounted for 470 ml ± 24 ml.

Based on these findings we expect a small standard derivation in body mass change on our test days as well.

Furthermore, the difference between weight loss (water loss) between the two test days is expected to be high, since sweat production in young children on a day with neutral temperatures is expected to be low [22–24].

The study named above [20] included 8 prepubertal male volunteers. Based on this study and considering the feasibility in a hectic kindergarten-day the number of participants was set at 30 subjects [20]. This sample size is estimated to be adequate to examine our hypothesis.

The statistical analysis in the planned study will be performed using the statistical software package IBM^®^ SPSS^®^ Statistics for Windows, version 29.0 (IBM Corp., Armonk, N.Y., USA). The significance level will be set at 0.05. For analyzing the primary hypothesis, a t-test for paired samples will be conducted. Since the same subjects will participate in day one as well as on day two, we obtain dependent datasets, which enable an intra-individual comparison. Further multivariate analyses will be performed using a general linear model with repeated measures or a multivariate Wilcoxon test.

## Discussion

The existence of the climate change is well researched [1]. In accordance with the position of the WHO, which elaborated aims for environmental health as well as sustainable development goals [25], facing public health issues in the upcoming years will be of increasing importance, especially those that affect children [18, 25, 26].

The current state of scientific knowledge on the potential long-term health effects of climate change on children is nevertheless insufficient. However, several potential physical health impacts are expected, including, besides of the potential development of chronic respiratory illnesses or risk of allergies and cardiovascular diseases, primarily potential risks due to heat, as for example heat stress or heat strokes [18]. Besides the health impacts itself, the consequences for health systems worldwide are a public health issue with increasing importance [4, 27]. These facts accentuate the need for fast action.

Insufficient water intake in accordance with rising quantity of hot days adds to the risk of unfavorable health outcomes due to climate change. Consequently, adequate drinking recommendations in accordance with different climate scenarios are needed, especially for young children. Hence, generating detailed knowledge of the quantity of water loss due to heat is an important first step. This study therefore will firstly provide new data for facing a major global health issue. The one-armed observational study design allows an intra-individual comparison of the collected data. Hence, interindividual variation of basal sweating rates and potential height, age or body surface differences are less impacting on the main findings of this paper. Since we do not choose a study design with an intervention, we forego the inclusion of a control group with other participants. The fall-like day is considered to be the control-day with comfortable temperatures in comparison to the hot day with heat stress to increase statistical relevance of the findings. By choosing non-invasive but precise measurements this study provides adequate data for new drinking recommendations while interfering as little as possible with the subjects’ daily routine.

Secondly, the comparison of the already published water loss model calculation of a 3-year-old child and the real-life scenario might provide information about the transferability of the results. The model was used as a starting point for setting a temperature target for the planned study. The following approach might outline, whether the German weather reaches high temperatures similar to the model climate scenario, despite the different latitude-positions of Germany and Japan. In case the results of the water loss calculation can be transferred to German conditions, the calculated water loss adds to the knowledge about physical thermoregulation response for young children under the impact of very high temperatures. With the modeled data further drinking recommendations for even more extreme climate scenarios might be obtained.

We are aware of some limitations of our study approaches. Individual and age-dependent variation of our subjects may have an impact on the accuracy of the comparison to the single model. Nevertheless, changes in the child’s development are not expected due to short time interval between two measurement days. Due to unsettled weather conditions in Germany with fast changing weather conditions, it is realistic to have perfect measurement conditions within two weeks. The possibility was conducted by analyzing former weather data of the study site Bochum, Germany and will be added to the publication of the main results of this study. Furthermore, weather changes are well predictable with the available data. However, a climatic comparison based on two climate scenarios might not provide enough information for possible transferability assessment.

Another limitation is the lack of a control group, which is not exposed to high temperatures on the hot summer day. Due to the size of the included kindergarten, splitting the group of participants would decrease the sample size in the “hot-weather-group” and therefore diminish the significance of the main results. Additionally, the deviations due to physiological and behavioral differences between the two groups would increase, rather than differences due to weather conditions. Nevertheless, the observation of another group of children and the comparison of both groups and both weather scenarios would increase the options for the analysis. Furthermore, the addition of a control group would inhibit the impact of predictors which might affect results like differences in diet, clothing or sleep of the participants. These factors need to be considered in the analysis and proper discussed in the main publication. Additionally, it needs to be considered to repeat the study with more than one study site and added control groups.

We nevertheless believe the chosen design provides data precise enough to estimate physiological differences in thermoregulation in high temperature scenarios of the temperate climate in Germany, which also can be compared to the thermoregulation in the subtropical climate in Japan. Therefore, the data might identify, whether the model calculation is applicable to Germany as well.

### Expected results

This study constructs three hypotheses, of which some are phrased directed, based on current scientific state of knowledge.

It is well known, that higher temperatures lead to higher sweating rates in humans [5]. Therefore, the primary hypothesis is proposed directed and tested one-sided via t-test. It is expected, that the water loss under heat exposure is significantly higher than under exposure to neutral temperatures. The exact amounts of water deprivation due to sweat is nevertheless unknown, especially for children.

For consideration of the secondary hypothesis, the physiological thermoregulation difference between the first and the second test day can be compared to the differences between the first test day (hot day) and the modeled results, since temperature differences will we approximately equal. In case the thermoregulatory responses are comparable the predicted water loss of the modeled calculation conclusively might indicate a further reference point for thermoregulatory in hotter environments than on the test day.

Due to expected higher water loss at higher ambient temperatures, it is expected that the change in hydration status on the hot day is higher than at the day with neutral temperatures. Voluntary drinking is expected to be little, since children will be distracted by outside playtime.

The findings of the other secondary hypothesis can reinforce the need for adequate drinking recommendations in accordance to different weather scenarios. Furthermore, the voluntary drinking amount might underline the need for early nutritional education and rising the awareness in parents, teachers and other caregivers.

Findings of this research project can contribute to knowledge of real water demand depending on the climate in the vulnerable group of young children. Updated drinking recommendations based on different weather scenarios can prevent heat-related illnesses and therefore contribute to protecting especially the affected youth. To communicate these updated recommendations with potential multipliers as for example kindergarten teachers, other educators or parents might raise awareness for the most important nutrient, water. Along with recommendations to prefer tap water as an environmentally friendly water source, these updated recommendations might as well contribute to sustainable development goals. This study therefore provides first important data. Nevertheless, further research is highly needed. In accordance with other findings above named issues can might be faced in the future.

## Data Availability

No datasets were generated or analysed during the current study.

## References

[1] Lee H, Calvin K, Dasgupta D, Krinner G, Mukherji A, Thorne PW, et al. Climate change 2023: synthesis report. Contribution of working groups I, II and III to the sixth assessment report of the intergovernmental panel on climate change. Geneva, Switzerland: Intergovernmental Panel on Climate Change (IPCC);: IPCC; 2023.

[2] Nazarian N, Krayenhoff ES, Bechtel B, Hondula DM, Paolini R, Vanos J, et al. Integrated assessment of urban overheating impacts on human life. Earths Future. 2022. 10.1029/2022EF002682.

[3] Ebi KL, Capon A, Berry P, Broderick C, de Dear R, Havenith G, et al. Hot weather and heat extremes: health risks. Lancet. 2021;398:698–708. 10.1016/S0140-6736(21)01208-3. 34419205 10.1016/S0140-6736(21)01208-3

[4] Choudhary E, Vaidyanathan A. Heat stress illness Hospitalizations — Environmental public health tracking Program, 20 States, 2001–2010. Surveillance Summaries. 2014;63;1–10. 25504077

[5] Cramer MN, Gagnon D. Human temperature regulation under heat stress in health, disease, and injury. Physiol Rev. 2022;102:1907–89. 10.1152/physrev.00047.2021. 35679471 10.1152/physrev.00047.2021 PMC9394784

[6] Kalhoff H, Sinningen K, Belgardt A, Kersting M, Luecke T. Climate change and fluid status in children: early education as one response to an emerging public health problem. Public Health Nutr. 2023;26:2891–4. 10.1017/S1368980023002562. 37981836 10.1017/S1368980023002562 PMC10755392

[7] Manz F, Wentz A, Sichert-Hellert W. The most essential nutrient: defining the adequate intake of water. J Pediatr. 2002;141:587–92. 10.1067/mpd.2002.128031. 12378203 10.1067/mpd.2002.128031

[8] Kalhoff H, Hilbig A, Libuda L. Trinken- was und Wie viel? Kinder- Und Jugendmedizin. 2015. 10.1055/s-0038-1629248.

[9] Rush EC, Chhichhia P, Kilding AE, Plank LD. Water turnover in children and young adults. Eur J Appl Physiol. 2010;110:1209–14. 10.1007/s00421-010-1621-5. 20734057 10.1007/s00421-010-1621-5

[10] Drozdowska A, Falkenstein M, Jendrusch G, Platen P, Luecke T, Kersting M, et al. Water consumption during a school day and children’s short-term cognitive performance: the CogniDROP randomized intervention trial. Nutrients. 2020. 10.3390/nu12051297. 32370147 10.3390/nu12051297 PMC7282257

[11] Kenney EL, Long MW, Cradock AL, Gortmaker L. Prevalence of inadequate hydration among US children and disparities by gender and race/ethnicity: national health and nutrition examination survey, 2009–2012. Am J Public Health. 2015;105:e113–8. 10.2105/AJPH.2015.302572. 26066941 10.2105/AJPH.2015.302572 PMC4504329

[12] Notley SR, Akerman AP, Meade RD, McGarr GW, Kenny GP. Exercise thermoregulation in prepubertal children: a brief methodological review. Med Sci Sports Exerc. 2020;52:2412–22. 10.1249/MSS.0000000000002391. 32366798 10.1249/MSS.0000000000002391 PMC7556246

[13] Kamiya T, Onishi R, Kodera S, Hirata A. Estimation of time-course core temperature and water loss in realistic adult and child models with urban micrometeorology prediction. Int J Environ Res Public Health. 2019. 10.3390/ijerph16245097. 31847195 10.3390/ijerph16245097 PMC6950469

[14] Hirata A, Asano T, Fujiwara O. FDTD analysis of body-core temperature elevation in children and adults for whole-body exposure. Phys Med Biol. 2008;53:5223–38. 10.1088/0031-9155/53/18/025. 18728308 10.1088/0031-9155/53/18/025

[15] Robert-Koch-Institut, editor. 4 Körpergewicht. In: Robert Koch-Institut, editor: Beiträge zur Gesundheitsberichtserstattung des Bundes. Referenzperzentile für anthropometrische Maßzahlen und Blutdruck aus der Studie zur Gesundheit von Kindern und Jugendlichen in Deutschland (KiGGS) [Contributions to federal health reporting. Reference percentiles for anthropometric measurements and blood pressure from the German Health Interview and Examination Survey for Children and Adolescents (KiGGS) ]. Berlin: Robert Koch-Institut; 2013. 2nd ed. p.22-31.

[16] Deutsche Gesellschaft für Ernährung e.V. (DGE). Wasser: Referenzwert. 2000. https://www.dge.de/wissenschaft/referenzwerte/wasser/. Accessed 8 Mar 2024.

[17] Iglesia I, Guelinckx I, de Miguel-Etayo PM, González-Gil EM, Salas-Salvadó J, Kavouras SA, et al. Total fluid intake of children and adolescents: cross-sectional surveys in 13 countries worldwide. Eur J Nutr. 2015;54(Suppl 2):57–67. 10.1007/s00394-015-0946-6. 26081646 10.1007/s00394-015-0946-6 PMC4473088

[18] UNICEF. UNICEF. The coldest year of the rest of their lives: protecting children from the escalating impacts of heatwaves. New York: UNICEF; 2022.

[19] Bar-David Y, Urkin J, Landau D, Bar-David Z, Pilpel D. Voluntary dehydration among elementary school children residing in a hot arid environment. J Hum Nutr Diet. 2009;22:455–60. 10.1111/j.1365-277X.2009.00960.x. 19486262 10.1111/j.1365-277X.2009.00960.x

[20] Inbar O, Morris N, Epstein Y, Gass G. Comparison of thermoregulatory responses to exercise in dry heat among prepubertal boys, young adults and older males. Exp Physiol. 2004;89:691–700. 10.1113/expphysiol.2004.027979. 15328309 10.1113/expphysiol.2004.027979

[21] Kojima K, Hirata A, Hasegawa K, Kodera S, Laakso I, Sasaki D, et al. Risk management of heatstroke based on fast computation of temperature and water loss using weather data for exposure to ambient heat and solar radiation. IEEE Access. 2018;6:3774–85. 10.1109/ACCESS.2018.2791962.

[22] Shibasaki M, Inoue Y, Kondo N, Aoki K, Hirata K. Relationship between skin blood flow and sweating rate in prepubertal boys and young men. Acta Physiol Scand. 1999;167:105–10. 10.1046/j.1365-201x.1999.00597.x. 10571545 10.1046/j.1365-201x.1999.00597.x

[23] Qingqing W, Jianhua L, Liang Z, Jiawen Z, Linlin J. Effect of temperature and clothing thermal resistance on human sweat at low activity levels. Build Environ. 2020;183:107117. 10.1016/j.buildenv.2020.107117.

[24] Taylor NA, Machado-Moreira CA. Regional variations in tranepidermal water loss, eccrine sweat gland density, sweat secretion rates and electrolyte composition in resting and exercising humans. Extrem Physiol Med. 2013. 10.1186/2046-7648-2-4. 23849497 10.1186/2046-7648-2-4 PMC3710196

[25] World Health Organization (WHO). Sustainable Development Goals. 11.03.2024. https://www.who.int/europe/about-us/our-work/sustainable-development-goals. Accessed 11 Mar 2024.

[26] World Health Organization. Putting children at the centre of the Sustainable Development Goals. 2020.

[27] Ahdoot S, Baum CR, Cataletto MB, Hogan P, Wu CB, Bernstein A, et al. Climate change and children’s health: builiding a healthy future for every child: policy statement. Pediatrics. 2024;153:75–85. 10.1542/peds.2023-065504 38374809

